# Comparative plastome analyses and evolutionary relationships of *Drynaria*

**DOI:** 10.3389/fpls.2025.1688693

**Published:** 2026-01-20

**Authors:** Xiao-Hua Chen, Jiang-Ping Shu, Juan Li, Yue-Hong Yan, Xi-Long Zheng, Yu-Feng Gu

**Affiliations:** 1 Key Laboratory of National Forestry and Grassland Administration for Orchid Conservation and Utilization, the National Orchid Conservation & Research Center of Shenzhen, Shenzhen, Guangdong, China; 2 School of Traditional Chinese Medicine, Guangdong Phamaceutical University, Guangzhou, Guangdong, China; 3 Fairy Lake Botanical Garden, Chinese Academy of Sciences, Shenzhen, Guangdong, China; 4 Shanghai Chenshan Plant Science Research Center, Chinese Academy of Sciences, Shanghai, China

**Keywords:** chloroplast genomes, *Drynaria*, evolution, fern, phylogeny

## Abstract

**Introduction:**

The genus *Drynaria*, a member of the Polypodiaceae family, exhibits substantial medicinal and ornamental value. Although molecular biological studies have elucidated the phylogenetic relationships in *Drynaria*, the characteristics of its plastome and the mechanisms underlying its adaptive evolution remain inadequately understood.

**Methods:**

This study performed a comprehensive comparative genomic analysis based on the plastomes of 15 *Drynaria* species. The research analyzed codon usage bias and identified positively selected genes within this genus. A well-supported phylogenetic tree was constructed using plastome data, and divergence times were measured at key nodes.

**Results:**

The analysis revealed that the plastomes of the 15 *Drynaria* species varied in size from 151,473 bp (*D. speciosa*) to 163,438 bp (*D. parishii*), each with 133 genes. Comparative analysis demonstrated conserved gene content, order, and orientation across all examined species, with no inversions or rearrangements except for a non-coding region rearrangement in the large single copy region of *D. roosii* and the small single copy region of *D. meyeniana*. Nucleotide diversity analysis identified seven hypervariable regions. The study detected 691 simple sequence repeats, 136 tandem repeats, and 750 dispersed repeats. Codon usage bias in *Drynaria* plastomes was predominantly influenced by natural selection. Phylogenetic reconstruction based on complete plastomes produced congruent topologies. Divergence time estimation suggested that *Drynaria* originated in the mid-Paleocene (59.75 Ma), with major diversification events occurring during the late Miocene (6–5 Ma). Selection pressure analysis revealed positive selection of *pet*A and *ycf*3 in branch models, while *ccs*A, *ycf*1, and *rpo*C2 exhibited evidence of positive selection in branch-site models.

**Discussion:**

These findings provide insights into the evolutionary adaptations and genomic features of this ecologically and economically significant fern genus.

## 1 Introduction

Soil functions as the primary source of nutrients for most plants, and its absence typically restricts plant growth. However, *Drynaria*, which attaches to tree trunks or rock surfaces (Zhang et al., 2013), demonstrates the ability to grow independently of soil. *Drynaria* species (Polypodiaceae family) form a substantial group of epiphytes; this genus includes approximately 50 species worldwide, distributed across tropical Africa, Indian Ocean islands, tropical Asia, tropical Australia, and Fiji (PPG I, 2016). In China, 13 species of *Drynaria* have been found in regions south of the Qingling Mountain (Qin, 1978; Zhang et al., 2013). Among these, *D. baronii*, *D. delavayi*, and *D. mollis* are endemic to China. The genus is distinguished by a robust, fleshy, creeping rhizome, drynarioid venation pattern, and humus-collecting leaves (Chen and Pang, 2012; Zhang et al., 2013; Yin, 2018). The morphological diversity of *Drynaria* species establishes them as an ideal group for studying epiphytes and provides a valuable model for understanding the mechanisms of biodiversity formation.

The plastome (chloroplast genome) demonstrates predominantly maternal inheritance, featuring a conserved structure, stable sequence composition, and moderate nucleotide substitution rate, establishing it as an essential resource in plant comparative genomics (Palmer, 1985; Palmer and Stein, 1986). Unlike the dynamic nuclear and mitochondrial genomes, the plastome’s structural stability enables robust cross-species comparisons to determine evolutionary relationships and species identification (Zhou et al., 2018; Liu et al., 2021; Huang et al., 2024; Wang et al., 2024). Shen et al. (2019) identified the *rbc*L gene and three additional sequences as diagnostic markers for distinguishing *Drynaria* species from related taxa to ensure the safe use of the traditional Chinese medicine “Gusuibu”. Moreover, plastomes have proven instrumental in revealing plant adaptive evolution mechanisms (Zeng et al., 2022; Yang et al., 2023; Xie et al., 2019). Selection pressure analyses of the sister mangrove species *Kandelia candel* and *K. obovata* revealed nonsynonymous mutations in *ndh*D and *atp*A genes. These mutations support divergent photosynthetic efficiency and energy synthesis, facilitating their adaptation to distinct geographic environments in the South China Sea (Xu et al., 2022). In addition, plastome data provide high-confidence scaffolds for reconstructing deep phylogenetic relationships and resolving taxonomic uncertainties in morphologically complex groups (Du et al., 2021; Wen et al., 2021; Xue et al., 2024) and are widely used in genetic diversity analysis and species identification (Song et al., 2023; Yang et al., 2018; Shen et al., 2025). Recent advances in Goodyerinae plastomics have revealed genus-specific structural features, such as inverted repeat (IR) boundary shifts and unique repeat configurations, which may facilitate genomic innovation and ecological adaptation (Tu et al., 2021). Therefore, comparative plastome analysis of *Drynaria* is essential for understanding its evolutionary trajectory, resolving phylogenetic conflicts, and uncovering mechanisms of environmental adaptation in epiphytic ferns.

We analyzed the plastome structure and composition of 15 *Drynaria* species, conducted phylogenetic reconstruction and divergence time estimation at the Polypodiaceae family level, and performed selection stress analysis on protein-coding genes. This comprehensive plastid genome analysis aims to (1) elucidate chloroplast-level adaptive evolution in *Drynaria*, (2) address phylogenetic relationships within the genus, and (3) trace its historical diversification patterns. We aim to prove the hypothesis that the current *Drynaria* is not a monophyletic group.

## 2 Materials and methods

### 2.1 Sample collection, plastome assembly, and annotation

Fresh leaf samples from 26 individuals representing 11 species were collected in Guangxi, Yunnan, Guizhou, and Tibet, with voucher specimens deposited at the Shenzhen Orchid Conservation Research Center (**Supplementary Table S1**). The leaves were dried and preserved in silica gel to prevent degradation before being sent to Novogene (Beijing, China) for sequencing. Paired-end sequencing was performed on an Illumina NovaSeq 6000 platform with 2 × 150 bp sequencing. The plastomes were assembled using GetOrganelle software (Jin et al., 2020), with *D. acuminata* (GenBank accession: NC_054156) serving as the reference. The assembled GFA files were visualized in Bandage (Wick et al., 2015) to verify the completeness of the plastome structure. When the GFA file did not form a complete circle, manual circularization was performed in Bandage using the FASTG format, or the assembly was repeated with SPAdes (Prjibelski et al., 2020). The genome was annotated using PAG (Qu et al., 2019), with *D. acuminata* (NC_054156) as the reference, and manually corrected in Geneious Primer (https://www.geneious.com) to ensure accurate gene start–stop codons, intron positions, and gene names.

### 2.2 Plastome feature analysis

The total length, large single copy (LSC), small single copy (SSC), and inverted repeat (IR) regions, along with gene composition, were analyzed using CPStools v2.0.2 (Huang et al., 2024). The GC content of the entire genome and its partitions (LSC, SSC, and IR) was calculated using Geneious Primer. Plastome maps were generated using the online tool OGDRAW (https://chlorobox.mpimp-golm.mpg.de/OGDraw.html).

### 2.3 Comparative analysis of plastomes

Global sequence alignment of *Drynaria* plastomes was conducted using mVISTA (https://genome.lbl.gov/vista/mvista/submit.shtml) in Shuffle-LAGAN mode. Synteny analysis was performed using the “Align Whole Genomes” feature of Geneious Primer. IRscope (https://irscope.shinyapps.io/irapp/) was used to examine IR region expansions or contractions. A sliding window analysis was executed with DnaSP (Rozas et al., 2017) to identify hypervariable regions and estimate nucleotide diversity (Pi). Sequences were aligned using MAFFT (Rozas et al., 2017), with a 200 bp step size and 600 bp window length.

Simple sequence repeats (SSRs) in the complete plastome were identified using MISA (Beier et al., 2017), applying thresholds of 8, 6, 5, 5, 5, and 5 repeats for mono- to hexanucleotide SSRs, with a minimum distance of 100 bp between SSRs. Long repeats were detected using REPuter (Kurtz et al., 2001) (Hamming distance = 3, max/min repeat size = 50/8). Tandem repeats were identified using Tandem Repeats Finder (Benson, 1999) with parameters: match = 2, mismatch = 7, delta = 7, PM/PI = 80/10, minimum alignment score = 50, and maximum repeat unit size = 500 bp. The data were processed in Excel.

### 2.4 Codon usage patterns

The GC content of each gene, including GC content at the first (GC1), second (GC2), and third (GC3) codon positions, the effective number of codons (ENC) and Relative synonymous codon usage (RSCU) values were calculated using CodonW v1.4.2 (Peden, 1999), and high-frequency codons (RSCU >1) were visualized using TBtools (Chen et al., 2023). For optimal codon analysis, genes with the highest and lowest 10% ENC values were designated as high- and low-expression libraries, respectively. ΔRSCU (RSCU_high_ − RSCU_low_) was calculated, and codons meeting RSCU >1 and ΔRSCU >0.08 were classified as optimal. PR2-plot analysis plotted A3/(A3+T3) against G3/(G3+C3). Neutrality plots regressed GC12 against GC3. ENC-plot analysis compared observed ENC values with expected values (ENC = 2 + GC3s + 29/[GC3s² + (1 − GC3s)²]).

### 2.5 Selection pressure analysis

Protein-coding genes shared among 15 *Drynaria* and 5 *Selliguea* plastomes were aligned using MAFFT (Katoh and Standley, 2013) in codon mode. Selection pressure analysis was performed using EasyCodeML (Gao et al., 2019), with the branch comprising all 15 *Drynaria* species designated as the foreground branch. Branch and branch-site models were used for detection. Likelihood ratio tests were performed to compare between branch models M1a and M2a as well as between branch-site models M7 and M8. Positively selected sites were identified after multiple-test correction using the QVALUE package, with a posterior probability threshold >0.95.

### 2.6 Phylogenetic analysis

To investigate the phylogeny of *Drynaria*, sequence data for 8 *Drynaria* species and 28 Polypodiaceae species were obtained from NCBI (**Supplementary Table S1**). A phylogenetic analysis was performed using 63 sequences, with species from the Loxogrammoideae family serving as the outgroup. Sequences were aligned using MAFFT (Katoh and Standley, 2013) and trimmed using trimAl (Capella-Gutiérrez et al., 2009). The best-fit model (GTR+F+I+G4) was determined using ModelFinder in PhyloSuite (Zhang et al., 2020). Maximum likelihood trees were constructed using IQ-TREE with 1000 bootstrap replicates (Nguyen et al., 2015), and Bayesian inference trees were generated using MrBayes with 1,000,000 generations, sampling every 1000th generation (Ronquist and Huelsenbeck, 2003). Convergence was evaluated (average standard deviation of split frequencies <0.01), and trees were visualized in FigTree (http://tree.bio.ed.ac.uk/software/figtree/).

### 2.7 Divergence time estimation

MCMCtree (dos Reis and Yang, 2011) within the PAML package was used to estimate divergence times within *Drynaria*, incorporating representative species from various Polypodiaceae subfamilies. Three fossil-based calibration points were established based on paleontological evidence and previous research: (1) the crown age of Polypodiaceae was adopted from Du et al.’s comprehensive divergence time estimation of Polypodiales using both PL and BEAST methods (age constraints of 71.17 Ma [minimum] and 79.38 Ma [maximum]); (2) the crown age of Goniophlebium was calibrated using the macrospore fossil *G. macrosorum* from the Middle Miocene deposits in Wenshan, Yunnan, China (age constraints of 15.2 Ma [minimum] and 16.5 Ma [maximum]); and (3) an internal node was calibrated using the *D. propinqua* leaf fossil found in the late Miocene Bangmai Formation strata in Lincang, Yunnan (age constraints of 5.3 Ma [minimum] and 11.6 Ma [maximum]). The analysis was performed by implementing MCMCtree with configured control parameters (mcmctree.ctl), using both time-calibrated rooted tree and multiple sequence alignment. The resulting chronograms were visualized and refined using FigTree software and the online visualization tool Chiplot (https://www.chiplot.online/), ensuring accurate representation of the temporal framework for *Drynaria* diversification. This comprehensive dating approach incorporated multiple independent fossil calibrations to provide robust estimates of evolutionary timescales within this ecologically significant fern lineage.

## 3 Results

### 3.1 Characteristics of *Drynaria* plastomes

**Figure 1:**
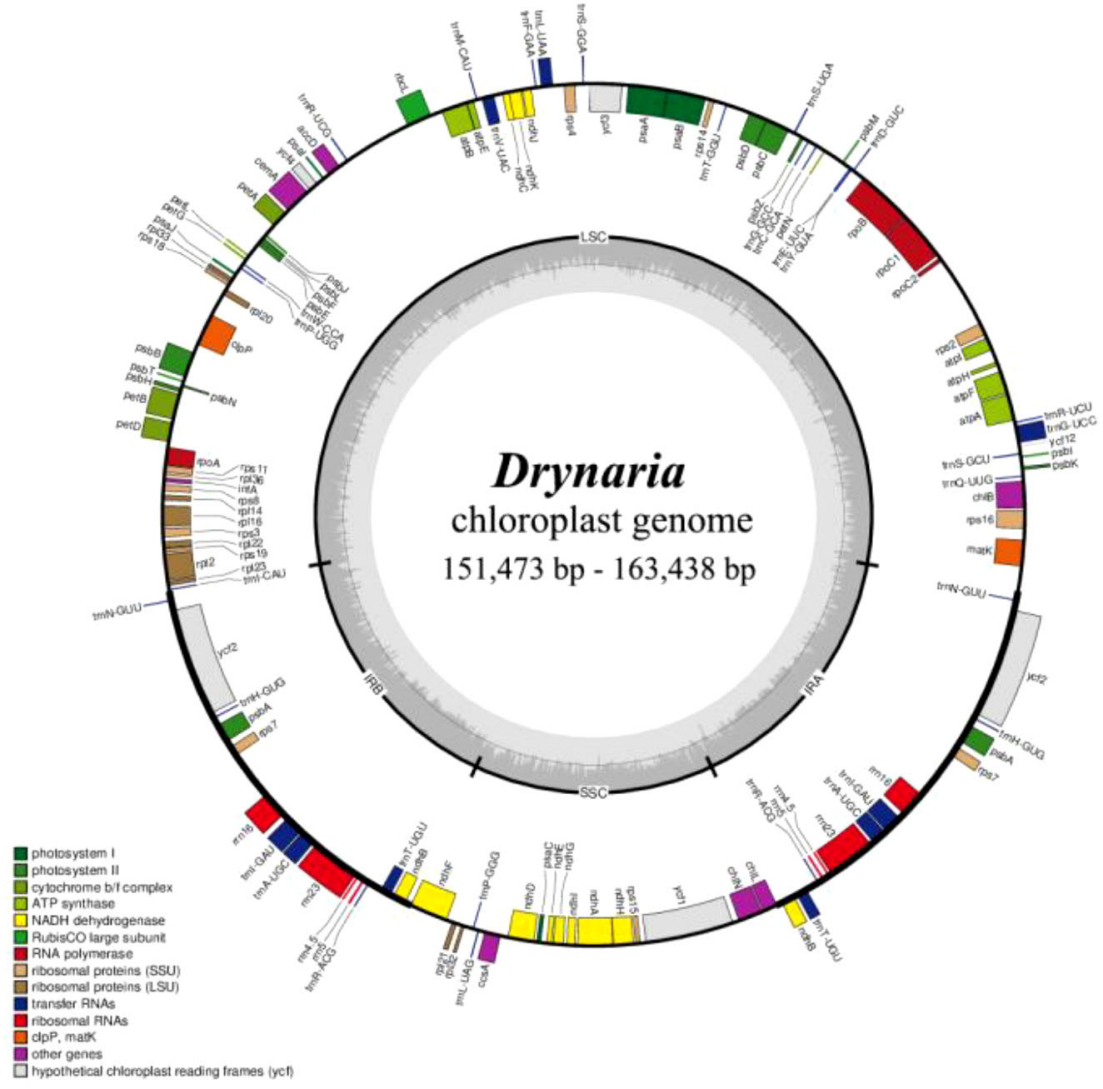
The comprehensive arrangement of the chloroplast genome in *Drynaria*. The large (LSC) and small (SSC) single copy regions are separated by the inverted repeats (IRa, IRb), represented by bold black lines on the inner circle. Genes located outside the circle undergo transcription in a counterclockwise fashion, while those inside undergo transcription in a clockwise direction.

We compared 15 species of *Drynaria* genus, those plastomes demonstrated a conserved quadripartite circular structure (**Figure 1**), with total lengths ranging from 151 to 162 kb and GC content of 40.8%–41.5%. While most species maintained typical region sizes of LSC (80–82 kb), SSC (21–22 kb), and IR (23–26 kb), three exceptions were observed: *D. coronans* and *D. parishii* exhibited IR expansion (27–30 kb), *D. roosii* displayed a longer LSC (85–86 kb), and *D. meyeniana* showed an extended SSC (24–25 kb) (**Supplementary Table S2**). All *Drynaria* plastomes encoded a conserved set of 133 genes, comprising 90 protein-coding, 4 rRNA, and 35 tRNA genes (**Supplementary Table S3**).

### 3.2 Sequence alignment, repeat sequence, SSR, and nucleotide diversity analyses

**Figure 2:**
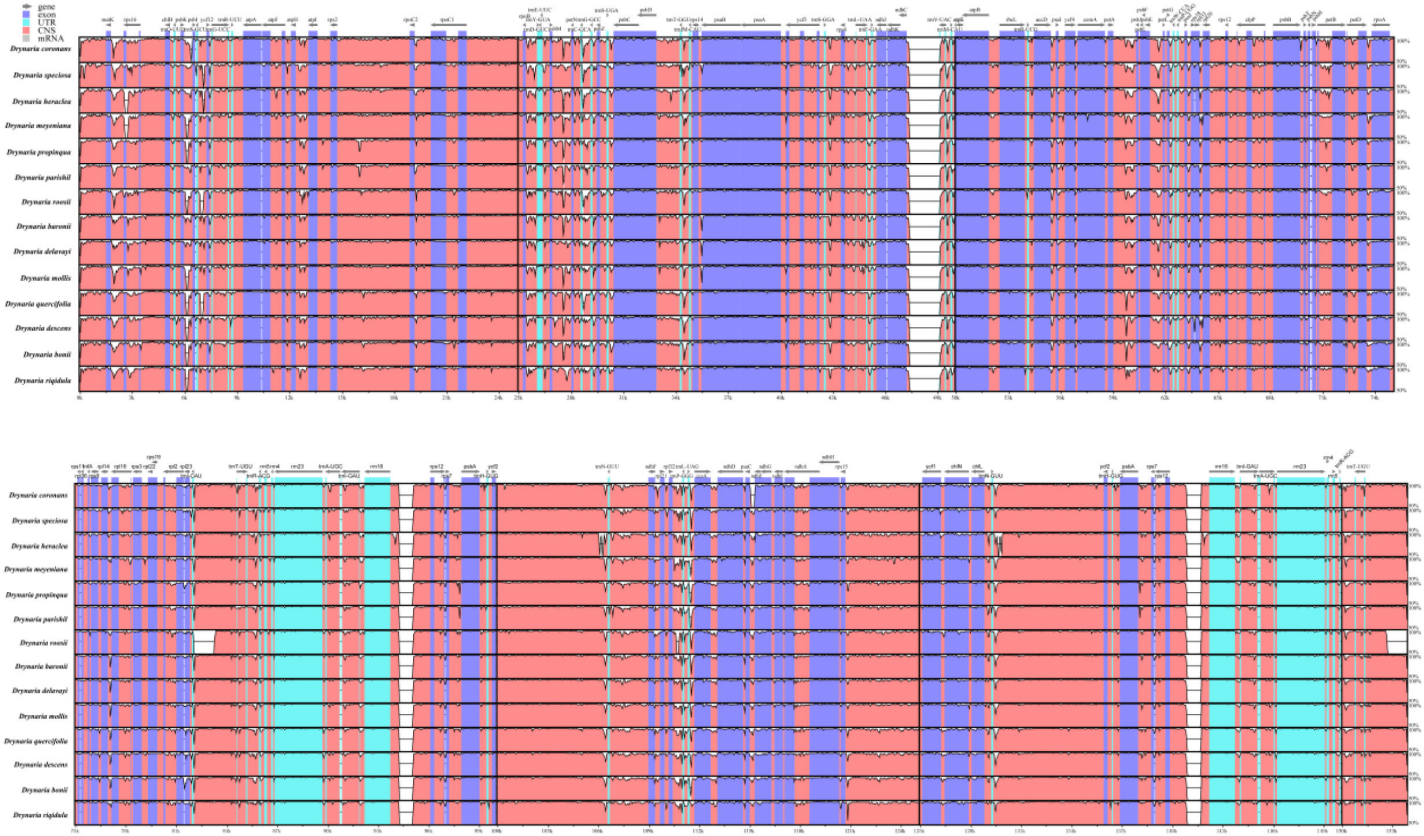
Comparison of 15 chloroplast genome of 15 *Drynaria* species. Gray arrows and thick black lines above the alignment indicate gene orientation. Exons are shown in dark blue. The red regions are Non-Coding Sequences. The light-blue regions are tRNA or rRNA. The vertical axis indicates the similarity among sequences, ranging from 50% to 100%.

Result of global alignment revealed high overall sequence conservation, with maximum variability localized in the LSC region, followed by the SSC, and minimal divergence in IRs. Comparative analysis indicated that intergenic spacers (non-coding sequences) exhibited significantly higher variability than coding sequences. While coding regions maintained uniformly high similarity, key non-coding regions, including *mat*K-*rps*16, *psb*K-*psb*I, *atp*H-*atp*I, *psb*M*-pet*N, *ndh*C-*trn*V-UAC, *pet*A-*psb*J, *rpl*23*-trn*T-UGU, and *rrn*16-*rps*12, displayed pronounced polymorphism and showed varying degrees of divergence. Compared with the other species, the *trn*I-CAU-*trn*T-UGU spacer in *D. roosii* exhibited substantial sequence divergence (**Figure 2**).

**Figure 3:**
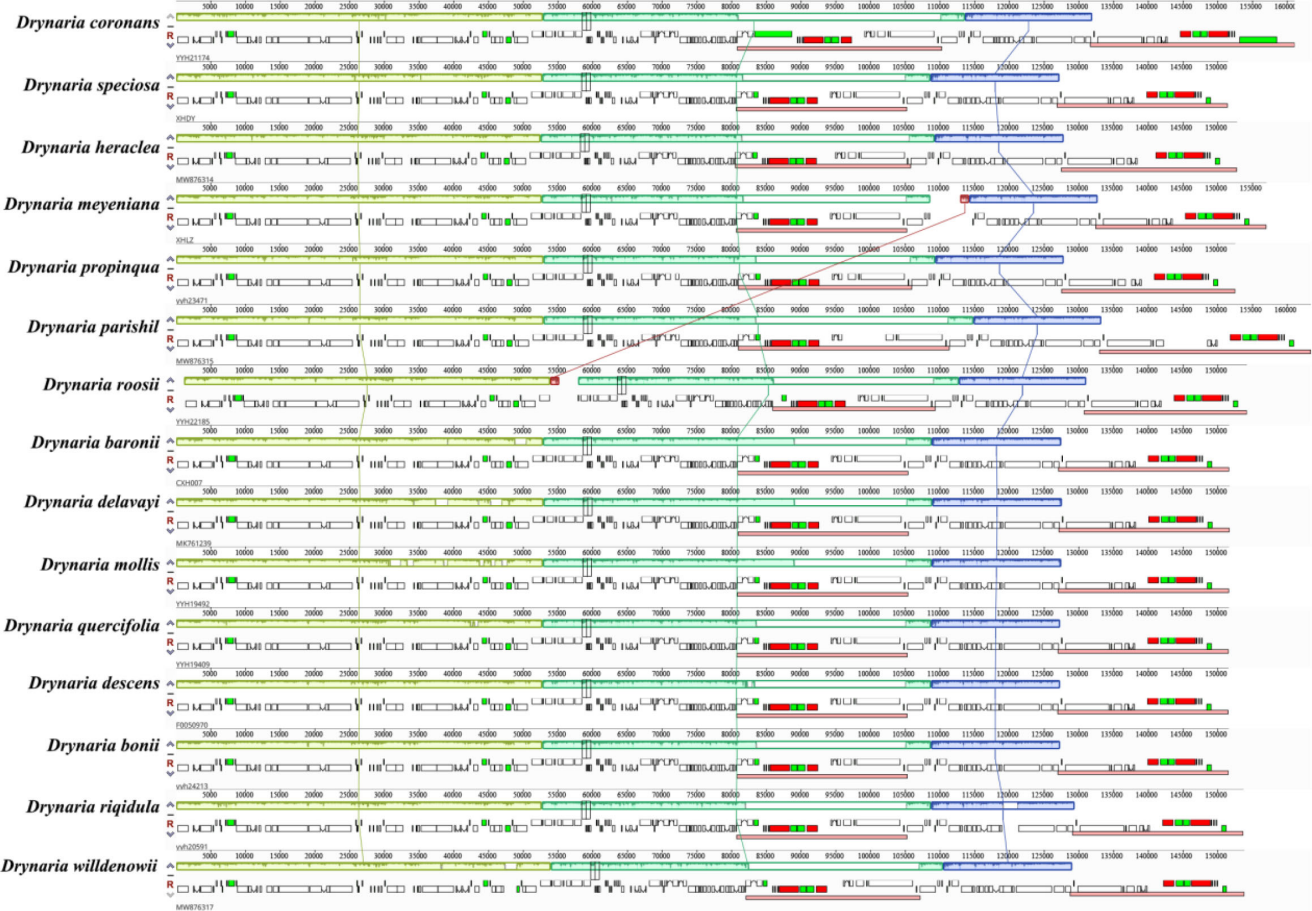
Mauve genome alignments of the whole chloroplast genomes of 15 *Drynaria* species. Different colors blocks represent Locally Collinear Blocks (LCBs) identified in *Drynaria*.

*Drynaria* species have three distinct locally collinear blocks, demonstrating conservation of gene content, copy number, and linear arrangement within the genus. However, comparative genomic analysis between *D. roosii* and *D. meyeniana* identified both unaligned regions and intersecting collinear blocks (highlighted in red), suggesting structural variations (**Figure 3**).

**Figure 4:**
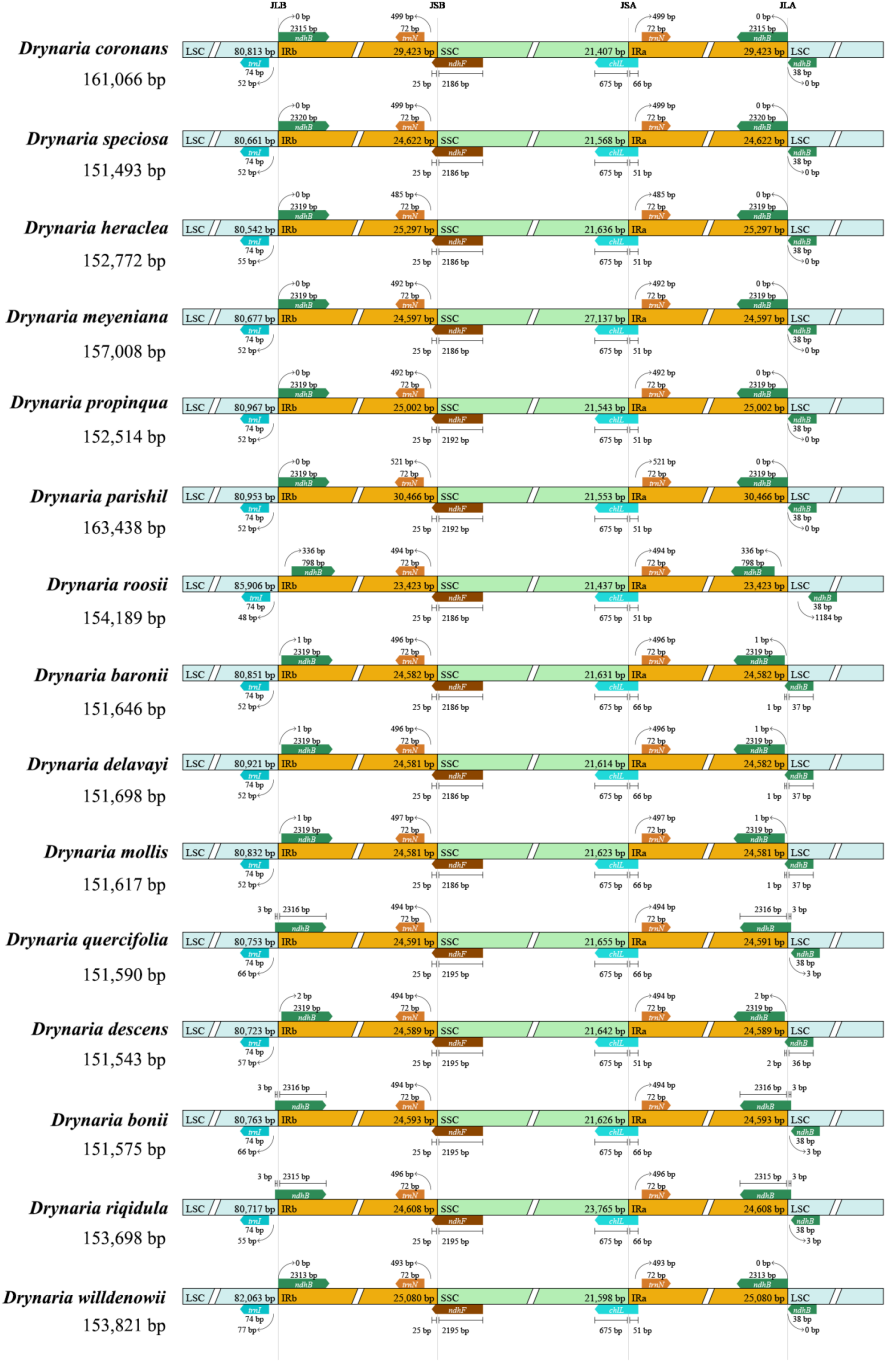
Comparison of boundaries regions of *Drynaria* chloroplast genome. Compare the boundaries of large orders (LSC), small single copies (SSC), and the border between the reverse repetition (lR) region.

Examination of four critical junction boundaries across 15 *Drynaria* species identified conserved flanking genes including *trnI*, *ndhB*, *trnN*, *ndhF*, and *chlL*, demonstrating remarkable evolutionary conservation of plastome architecture among *Drynaria* species. Minimal structural divergence and stable patterns of IR boundary expansion–contraction were observed. Minor variations specific to *D. roosii* indicate potential lineage-specific genomic modifications (**Figure 4**).

Nucleotide diversity (Pi) in *Drynaria* species ranged from 0.00022 to 0.10402, indicating high sequence conservation within the genus. The IR regions showed significantly lower average Pi values compared with both LSC and SSC regions, consistent with typical plastome evolution and confirming their enhanced evolutionary stability. The analysis identified seven highly variable regions with Pi values exceeding 0.05: the *ndh*C-*trn*M-CAU intergenic spacer, *rps*16 gene, *rpo*B*-trn*D*-*GUC spacer, *pet*A*-psb*J spacer, *rrn*16*-rps*12 spacer, *rps*12*-rrn*16 spacer, and *ndh*F gene fragment (**Supplementary Figure 1**).

Analysis of 15 *Drynaria* plastomes identified 691 SSRs, distributed as 369 (53.40%) in LSC, 204 (29.52%) in IR, and 118 (17.08%) in SSC. Additionally, 136 tandem repeat were detected, primarily in the LSC (58), followed in IR (34 each), and SSC (10). Tandem repeat numbers varied significantly among species, ranging from 20 in *D. parishii* to 4 in *D. mollis*, with SSC regions consistently having the lowest counts (1–4 repeats). Furthermore, 750 dispersed repeats (avg. 50 per genome) included complementary (344), reverse (219), palindromic (156), and forward (31) types. While most tandem repeats were 17–30 bp, *D. parishii* predominantly had longer 34–44 bp repeats (**Supplementary Figure 2**).

### 3.3 Codon usage bias analysis

**Figure 5:**
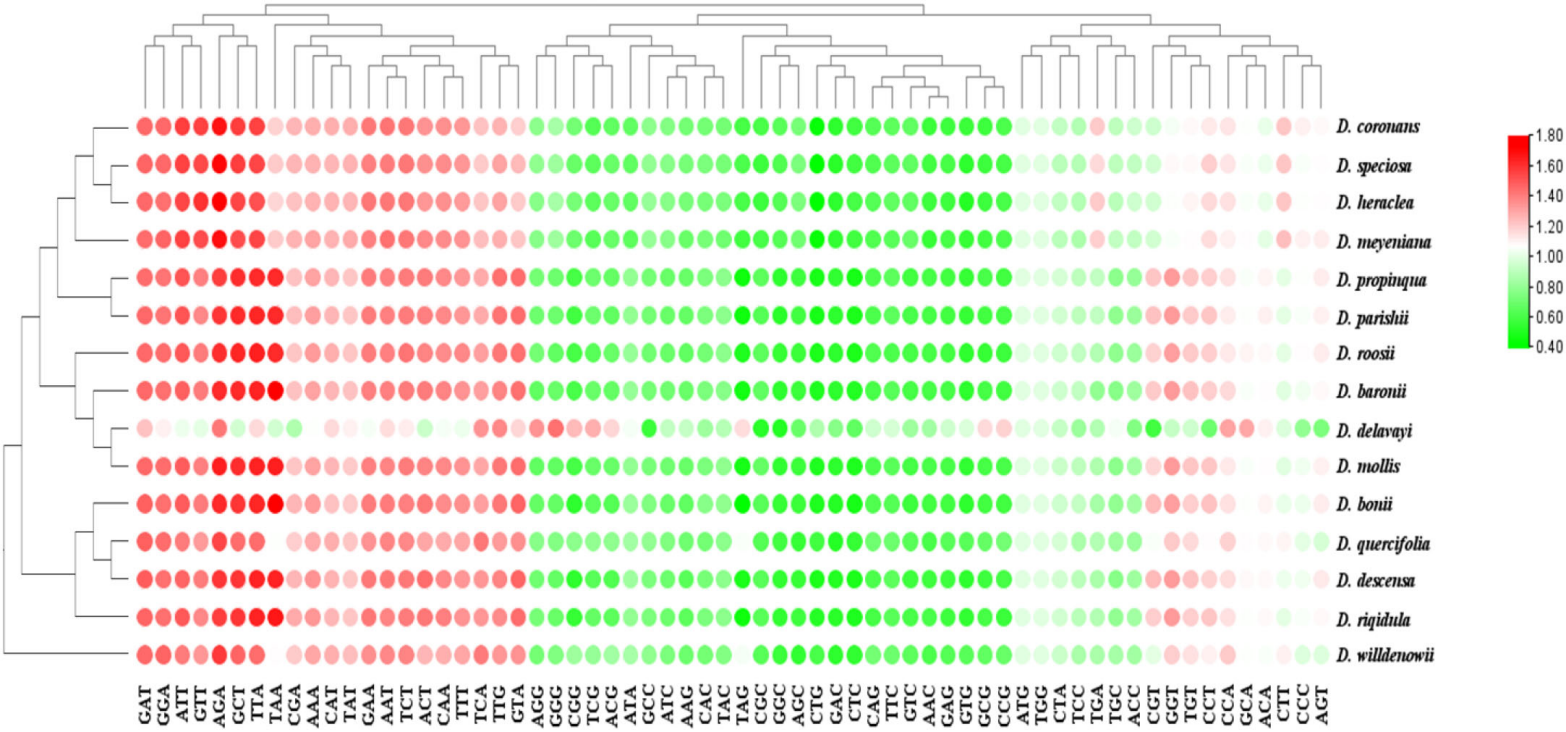
RSCU heat map of the Coding gene sequence of the 15 *Drynaria* species. Values are represented by a dual-color gradient: increasing values are shown in darker red, whereas decreasing values are shown in darker green.

RSCU values of *Drynaria* plastomes ranged from 0.54 to 1.84, with species containing 26–30 codons showing RSCU >1. *D. roosii* exhibited the highest number of preferred codons, while *D. coronans* showed the lowest. *D. roosii* primarily used AGA (Arg), UUA (Leu), and GCU (Ala), while other species favored GUU (Val), AUU (Ile), and AGA (Arg). Several GC-terminated codons demonstrated both high RSCU values (>1) and significant usage bias, including AGC (Ser), UGC (Cys), GGC (Gly), UUG (Leu), AGG (Arg), CCC (Pro), ACC (Thr), and UCC (Ser) (**Figure 5**).

PR2-plot analysis revealed an asymmetric distribution of *Drynaria* chloroplast genes, with most positioned away from the central (0.5, 0.5), demonstrating that codon usage bias was influenced by both natural selection and mutational pressure. *Drynaria* species exhibited preferential use of T over A and G over C at the third codon position. In ENC-plot analysis, most genes deviated considerably below the standard curve. This, combined with neutrality plots showing weak, non-significant positive correlations (regression coefficients: 0.17-0.31) between GC12 and GC3, collectively supports that natural selection was the dominant force shaping codon usage (**Supplementary Figure 3**).

### 3.4 Selection pressure analysis

To evaluate potential positive selection in *Drynaria*, comparative analyses of Figurprotein-coding sequences were conducted between 15 *Drynaria* species and 5 *Selliguea* species, designating *Drynaria* as the foreground branch. The branch model identified two genes (*pet*A and *ycf*3) exhibiting signatures of positive selection (**Table 1**), while the branch-site model identified three additional genes (*ccs*A, *ycf*1, and *rpo*C2) (**Table 2**). These results suggest adaptive evolution in chloroplast genes involved in photosynthesis (*pet*A, *ccs*A, and *rpo*C2) and genome maintenance (*ycf*1 and *ycf*3).

**Table 1:** Analysis of selection pressure based on branch model in *Drynaria*.

Gene	Model	ln L	LRT p value	ω value
*pet*A	Two ratio Model 2	-2399.982381	0.000000000	ω1 = 111.33716
*ycf*3	Two ratio Model 2	-897.979444	0.040587664	ω1 = 999.00000

**Table 2:** Analysis of selection pressure based on branch site model in *Drynaria*.

Gene	Model compared	LRT p value	Positive sites
*ccs*A	Model A vs.Model A null	0.000000000	159 V 0.581,160 L 0.539,184 E 0.573,191 C 0.513,192 N 0.583,303 S 0.582,306 L 0.567,347 K 0.571,443 S 0.554,456 N 0.581,460 S 0.530
*ycf*1	Model A vs.Model A null	0.000000000	30 K 0.911,37 T 0.911,103 H 0.909
*rpo*C2	Model A vs.Model A null	0.000000000	775 D 0.527

### 3.5 Phylogenetic analysis

**Figure 6:**
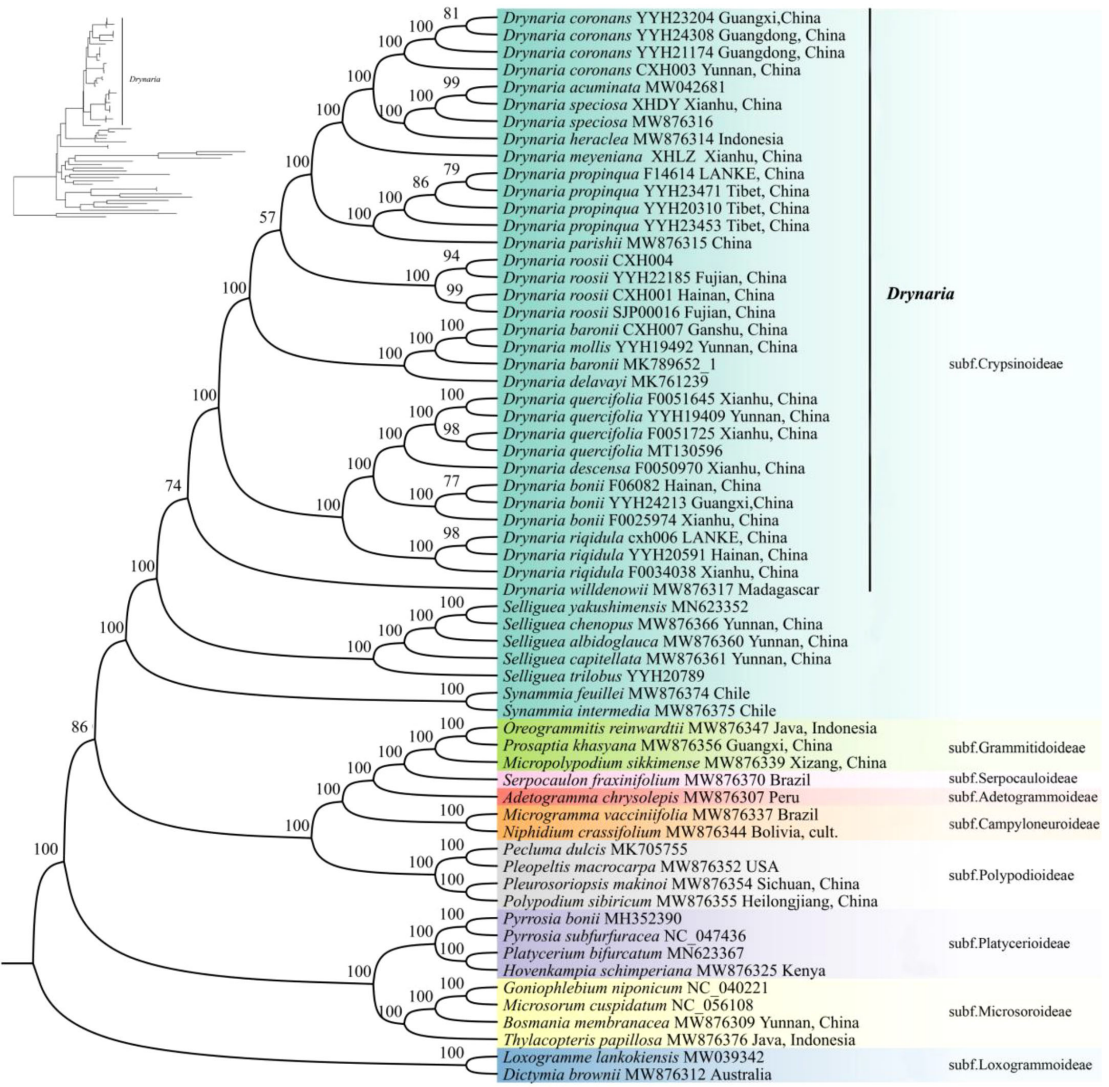
Phylogenetic relationships inferred from maximum likelihood based on CDS. Numbers above the branches are the bootstrap values.

Phylogenetic reconstruction of *Drynaria* using concatenated protein-coding genes and complete plastomes yielded congruent, well-supported topologies. The concatenated alignment of complete plastome sequences was 153,479 bp in length. Analyses placed *D. rigidula* as the earliest diverging lineage among Chinese taxa, forming a cluster with *D. quercifolia* and *D. bonii* that serves as sister to all other *Drynaria* species. Subsequent divergences formed distinct lineages: first *D. delavayi* and *D. baronii*, followed by *D. roosii*, then a *D. parishii*–*D. propinqua* clade, and finally a robust clade of *D. meyeniana, D.* sp*eciosa*, and *D. coronans* (**Figure 6**, **Supplementary Figure 4**).

### 3.6 Divergence time estimation

**Figure 7:**
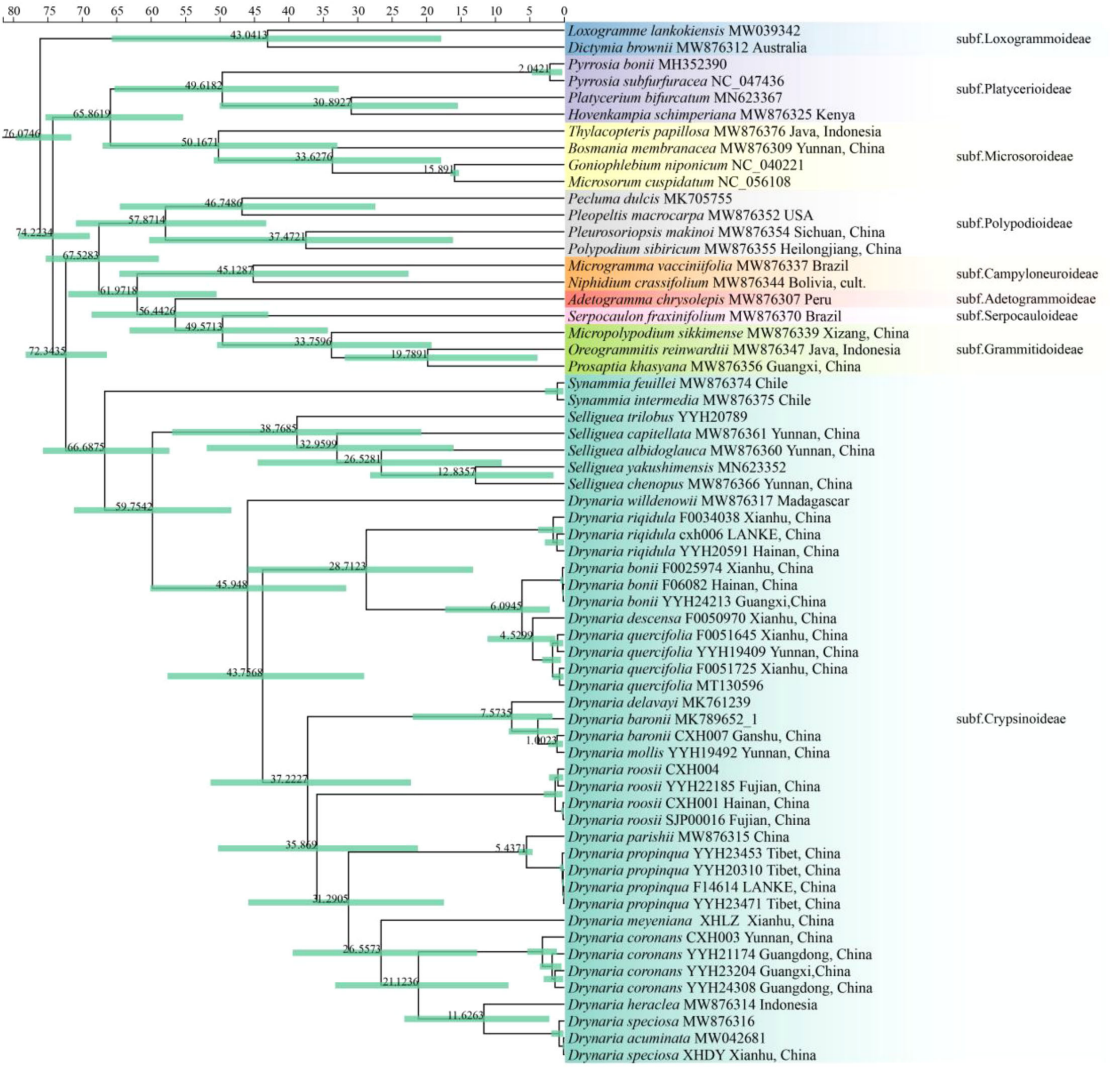
The maximum clade credibility tree of *Drynaria* was constructed using the MCMCtree method, based on the chloroplast genome sequences.

Molecular dating indicates that *Drynaria* diverged from its sister genus *Selliguea* approximately 59.75 Ma (95% HPD = 71.17–48.27 Ma) during the late Paleocene. Diversification within *Drynaria* initiated approximately 6–5 Ma, with major cladogenesis events occurring during the late Miocene. This temporal framework corresponds to periods of significant climatic change and tropical forest expansion, suggesting that adaptive radiation in these epiphytic ferns was facilitated by ecological opportunities arising from the development of recent tropical forest ecosystems (**Figure 7**).

## 4 Discussion

### 4.1 Structural variation in *Drynaria* plastomes

While plastomes generally exhibit more conservation than nuclear genomes, substantial genetic variation exists within chloroplast DNA, including SSRs, single-nucleotide polymorphisms, and insertions–deletions. Recent studies have identified structural rearrangements in various plant groups such as Cactaceae family and *Medicago* genus. Our comparative genomic analysis revealed conserved gene content and synteny across *Drynaria* species. However, significant structural variations were observed between *D. roosii* and *D. meyeniana*, particularly in non-coding regions with disrupted sequence microcollinearity. These disruptions indicate potential genome rearrangements that contain functionally important elements. The identification of abundant repetitive elements, including 691 SSRs, 136 tandem repeats, and 750 dispersed repeats in *Drynaria* plastomes, supports the hypothesis that repeats facilitate structural diversification. Notably, *D. roosii* demonstrated elevated repeat density in the LSC region, a pattern associated with localized genomic instability. Substantial length variation was identified in IR regions, with *D.* coronans and *D. parishii* showing 5–6 kb expansions compared with the other species. These expansions correlate with increased tandem repeat content (6–8 repeats vs. 1–3 in other species), suggesting repetitive elements as drivers of IR boundary dynamics.

### 4.2 DNA barcoding markers for species delineation

Our nucleotide diversity analysis identified multiple hypervariable regions suitable for species discrimination, specifically intergenic spacers (e.g., *rpo*B-*trn*D-GUC, *pe*tA-*psb*J and *rps*12-*rrn*16) and coding genes (*ndh*F and *rps16*). Of particular significance is the highly divergent *trn*I-CAU-*trn*T-UGU region in *D. roosii*, which functions as a reliable molecular marker distinguishing it from congeners. These findings extend previous work by Shen et al. (2019) and provide additional genomic resources for refining taxonomic delineations within *Drynaria*.

### 4.3 Adaptive evolution in *Drynaria*

Although both are epiphytic plants, species in the genus *Selliguea* have slender rhizomes, whereas those in the genus *Drynaria* have stout rhizomes. Also, *Drynaria* species exhibit distinctive ecological specialization and are primarily distributed in tropical and subtropical regions. This distribution pattern suggests the development of specialized adaptive responses to environmental conditions, with corresponding adaptive changes potentially detectable in their plastomes. The analysis revealed positive selection in two plastid genes (*pet*A and *ycf*3), indicating their potential contribution to environmental adaptation in *Drynaria*. The *pet*A gene, encoding a cytochrome *b_6_f* complex subunit, exhibited strong selection signals, potentially enhancing photosynthetic efficiency through optimized electron transport and conferring stress tolerance in these epiphytes (Cramer et al., 2011; Tikhonov, 2014). The gene *ycf*3 mediates the accumulation of the photosystem I complex. Branch-site models revealed additional selected genes (*ccs*A, *ycf*1, and *rpo*C2) involved in critical functions. The *ccs*A gene encodes a protein essential for the binding of hemoglobin to c-type cytochromes, facilitating heme attachment to these molecules (Xie and Merchant, 1996). *ccs*A is under positive selection in two epiphytic *Ficus* species, *F. aurea* and *F. cyathistipula* (Zhang et al., 2022). The *ycf*1 gene is associated with maintaining plastome stability (Drescher et al., 2000), and *rpo*C2 is involved in chloroplast transcription (Börner et al., 2015). These molecular adaptations likely underpin *Drynaria’*s ability to thrive in drought-prone, low-light epiphytic niches.

### 4.4 Phylogenetic relationships

Previous phylogenetic studies using molecular markers established the close relationships among *Drynaria*, *Aglaomorpha*, *Pseudodrynaria*, and *Thayeria* within Polypodiaceae (Schneider et al., 2004a, 2004b; Janssen and Schneider, 2005). Subsequent morphological analysis of clathrate rhizome scales confirmed *Christiopteris* as a member of *Drynaria* (Schneider et al., 2008). The current phylogenomic reconstruction, based on 34 plastomes from 15 *Drynaria* species, strongly corroborates these findings and supports the taxonomic merger of *Drynaria* and *Aglaomorpha*, aligning with recent classifications (Schneider et al., 2008; PPG I, 2016; Wei and Zhang, 2022). While Chandra’s morphological comparisons of sporophytes suggested that the genus *Drynaria* was derived from *D. rigidula* (Chandra, 1981), our phylogenetic results support the placement of *D. willdenowii* as the basal lineage of the genus. Although traditional morphological studies considered *D. descensa* to be a derived form of *D. quercifolia* (Copeland, 1960; Chandra, 1981)—a view consistent with their sister relationship in our phylogeny—our molecular data unexpectedly place the divergence of *D. descensa* before that of *D. rigidula* (posterior probability >0.95). This indicates that the evolutionary history of this lineage is more complex than previously inferred from morphological evidence alone. This discrepancy potentially reflects either rapid morphological evolution in *D*. *descensa*, leading to convergent traits with *D. quercifolia*, or insufficient sampling, particularly for early *Drynaria* lineages. The three species *D. baronii, D. delavayi, and D. mollis*—all endemic to China and restricted to high-altitude areas (e.g., Yunnan, Tibet, Gansu, Qinghai)—form a monophyletic clade in our phylogeny. This phylogenetic grouping potentially reflects their shared evolutionary history in these isolated habitats. These results demonstrate the value of genome-scale data for resolving challenging phylogenetic relationships in this ecologically rapidly radiating fern group.

### 4.5 Divergence times

Molecular dating establishes the origin of *Drynaria* in the mid-Paleocene (59.75 Ma, 95% HPD: 71.17–48.27 Ma). The prevailing global greenhouse conditions during this warm climatic period likely facilitated its initial diversification, suggesting a thermophilic origin for this fern lineage.

The documented fossil records of *Drynaria* in China date back to the late Miocene to late Pliocene epoch. Paleontological evidence strongly supports this Neogene period as a critical phase of diversification within the genus (Su et al., 2011; Wu et al., 2012; Wen et al., 2013; Huang et al., 2016), aligning with the estimated timeframe (6–5 Ma) for the radiation of *Drynaria* species diversity.

By integrating structural genomics, molecular evolution, and phylogenetic dating, this study illuminates the genomic basis of *Drynaria*’s ecological success—particularly its adaptations to nutrient acquisition in canopy habitats (e.g., humus-collecting structures documented since the Pliocene) and drought resilience in variable microclimate. These findings establish a comprehensive evolutionary timeline for *Drynaria*’s diversification and specialization in epiphytic niches within tropical forest ecosystems.

## Data Availability

The datasets presented in this study can be found in online repositories. The names of the repository/repositories and accession number(s) can be found in the article/**Supplementary Material**.
