# Supplementary material for: Comparative plastome analyses and evolutionary relationships of Drynaria
Source: Front Plant Sci. 2026 Jan 20;16:1688693. doi: 10.3389/fpls.2025.1688693 (PMC12864413; doi:10.3389/fpls.2025.1688693)
Supplement: Supplementary file 1 [file DataSheet1.docx]

# Supplementary Figures and Tables

## Supplementary Figures

## Supplementary Figure 1. Codon bias analysis of chloroplast genomes of 15 species of *Drynaria*


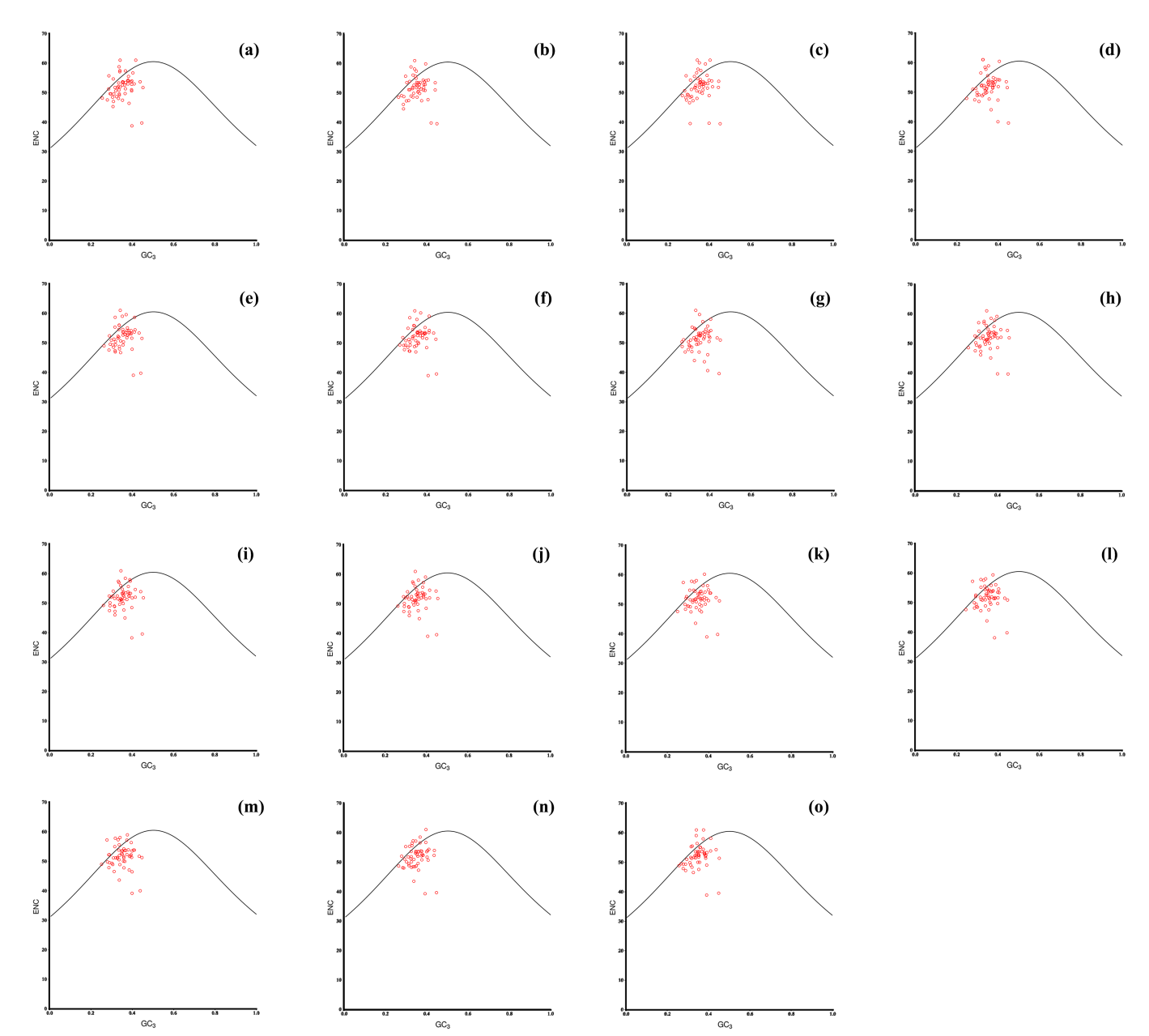


ENC-plot analysis of chloroplast genomes of 15 species of *Drynaria* s.l. (a) *D. coronans*；(b) *D. speciosa*；（c）*D. heraclea*；(d) *D. popinqua*；(e) *D. meyeniana*；(f) *D. parishii*；(g) *D. roosii*；(h) *D. baronii*；(i) *D. mollis*；(j) *D.quercifolia*；(k) *D. descensa*；(l) *D. bonii*；(m) *D. rigidula*；(o)*D. willdenowi*


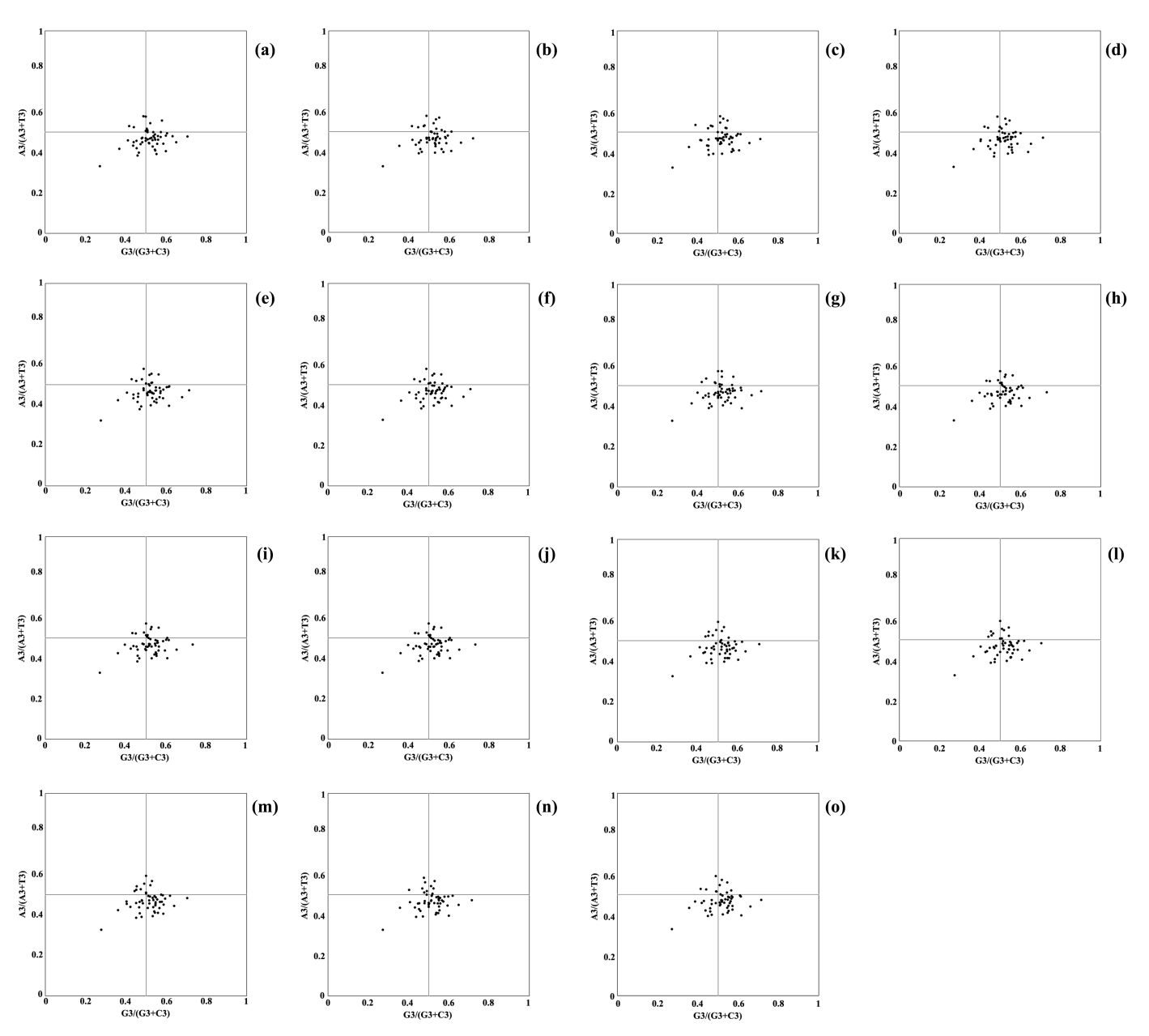


PR2-plot analysis of chloroplast genomes of 15 species of *Drynaria* s.l. (a) *D. coronans*；(b) *D. speciosa*；（c）*D. heraclea*；(d) *D. popinqua*；(e) *D. meyeniana*；(f) *D. parishii*；(g) *D. roosii*；(h) *D. baronii*；(i) *D. mollis*；(j) *D.quercifolia*；(k) *D. descensa*；(l) *D. bonii*；(m) *D. rigidula*；(o)*D. willdenowi*

##
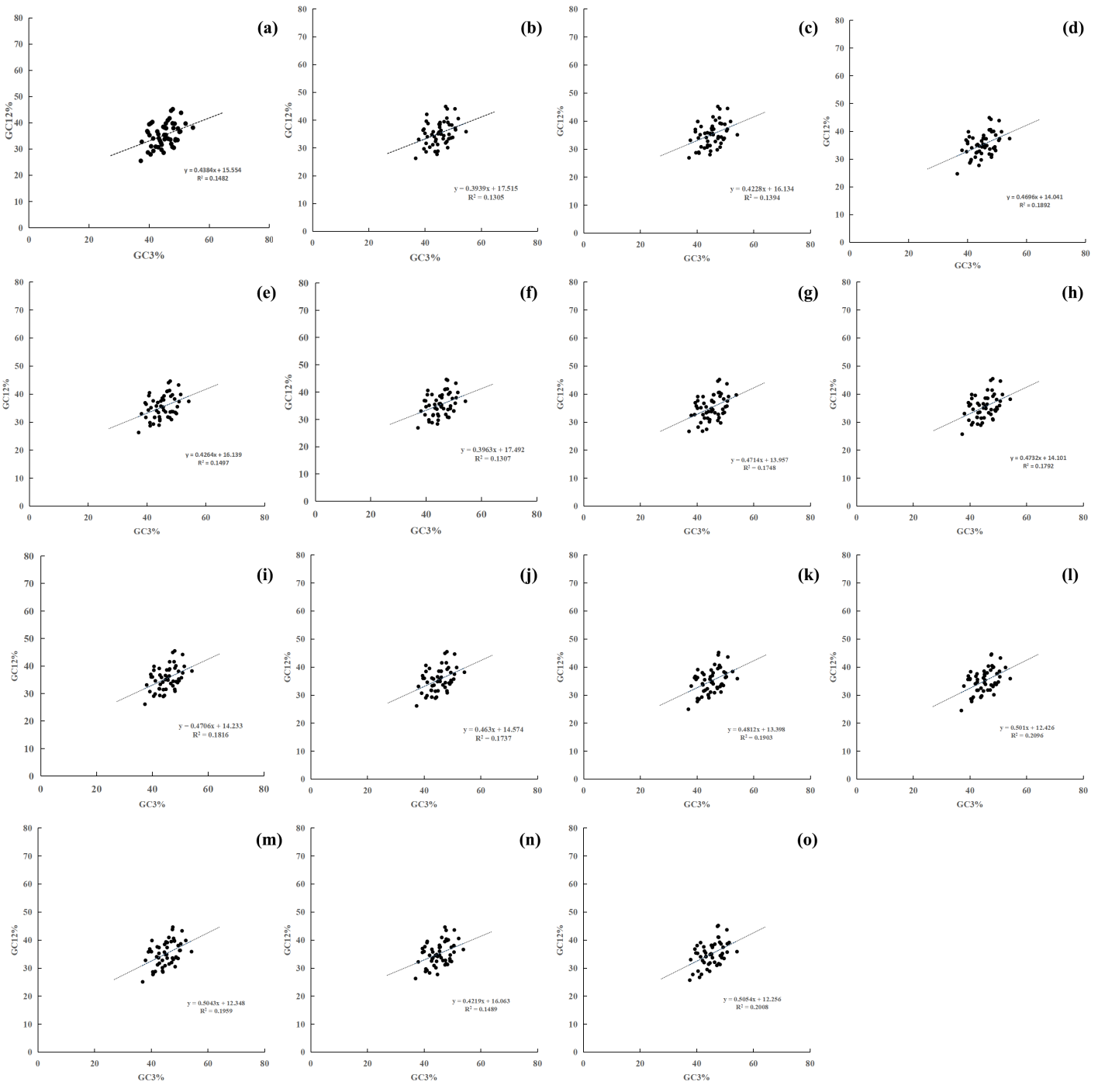


Neutral plot analysis of chloroplast genomes of 15 species of *Drynaria* s.l. (a) *D. coronans*；(b) *D. speciosa*；（c）*D. heraclea*；(d) *D. popinqua*；(e) *D. meyeniana*；(f) *D. parishii*；(g) *D. roosii*；(h) *D. baronii*；(i) *D. mollis*；(j) *D.quercifolia*；(k) *D. descensa*；(l) *D. bonii*；(m) *D. rigidula*；(o)*D. willdenowi*

## Supplementary Figure 2. The construction of Bayesian phylogenetic trees based on the chloroplast genome.

*
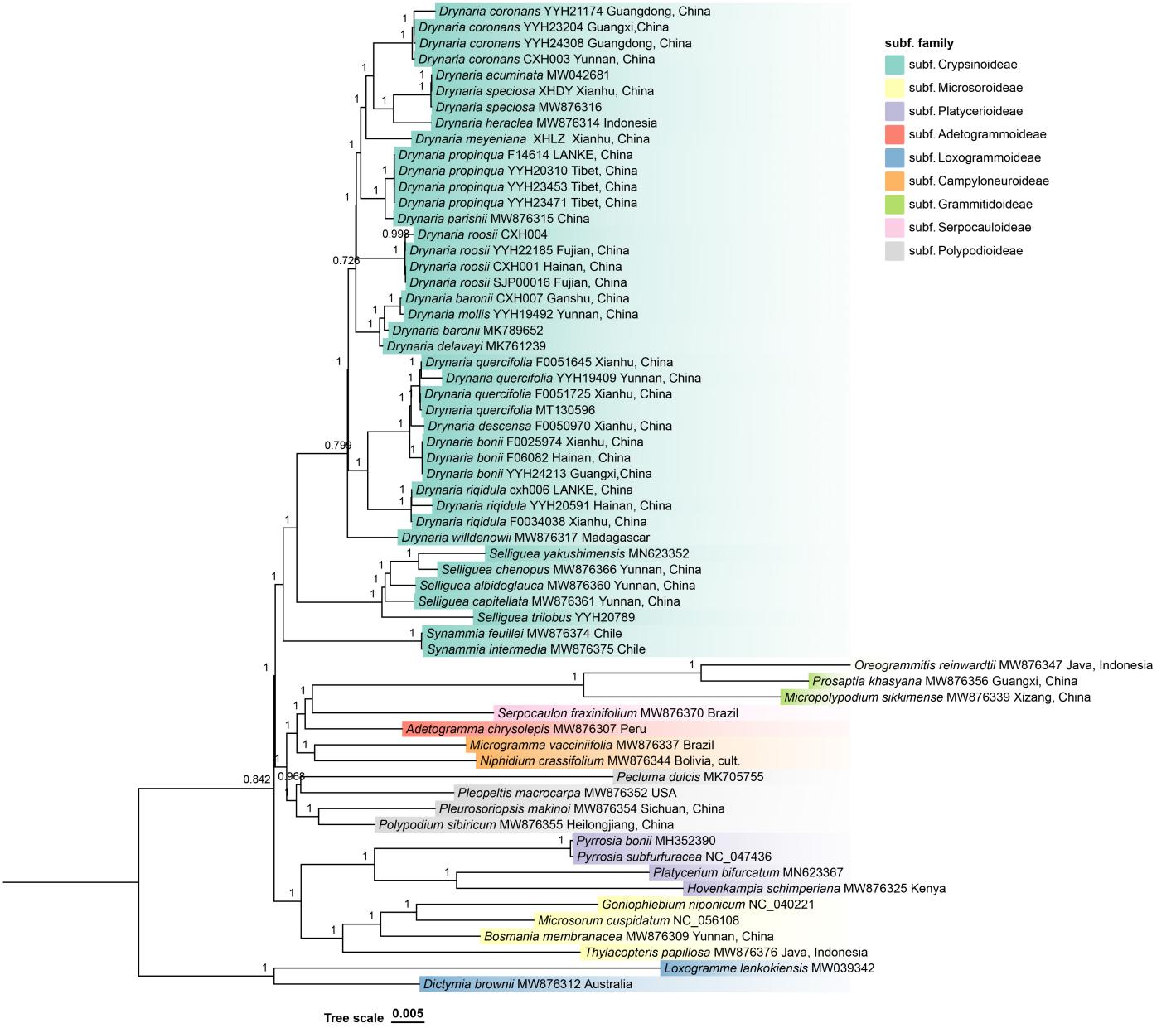
*

## Supplementary Table

## Supplementary Table S1． Information of all the employed samples, * were new sequencing for this study, - were missing data.

| Subfamily name | Species | Locality | No. |
| --- | --- | --- | --- |
| Adetogrammoideae | Adetogramma chrysolepis (Hook.) T.E.Almeida | China, Yunnan, R. Wei WR0368 | MW876307 |
| Campyloneuroideae | Microgramma vacciniifolia (Langsd. & Fisch.) Copel. | Brazil, C.X. Li RS281 | MW876337 |
| Campyloneuroideae | Niphidium crassifolium (L.) Lellinger | Bolivia, cult. in Beijing, R. Wei WR0621 | MW876344 |
| Crypsinoideae | Drynaria acuminata Fée | China, Yunnan, Liu-CP13 | MW042681 |
| Crypsinoideae | Drynaria baronii Diels | China, Ganshu, CXH007 | *C_AA120336.1 |
| Crypsinoideae | Drynaria baronii Diels | - | MK789652 |
| Crypsinoideae | Drynaria bonii Christ | China, Xianhu, F0025974 | *C_AA120337.1 |
| Crypsinoideae | Drynaria bonii Christ | China, Hainan, F06082 | *C_AA120342.1 |
| Crypsinoideae | Drynaria bonii Christ | China, Guangxi, YYH24213 | *C_AA120359.1 |
| Crypsinoideae | Drynaria coronans (Wall. ex Mett.) J.Sm. | China, Yunnan, CXH003 | *C_AA120334.1 |
| Crypsinoideae | Drynaria coronans (Wall. ex Mett.) J.Sm. | China, Guangdong, YYH21174 | *C_AA120349.1 |
| Crypsinoideae | Drynaria coronans (Wall. ex Mett.) J.Sm. | China, Guangxi, YYH23204 | *C_AA120351.1 |
| Crypsinoideae | Drynaria coronans (Wall. ex Mett.) J.Sm. | China, Guangdong, YYH24308 | *C_AA120352.1 |
| Crypsinoideae | Drynaria delavayi Christ | - | MK761239 |
| Crypsinoideae | Drynaria descensa Copel. | China, Xianhu, F0050970 | *C_AA120339.1 |
| Crypsinoideae | Drynaria heraclea (Kunze) T.Moore | Indonesia, Java, R. Wei 470 | MW876314 |
| Crypsinoideae | Drynaria meyeniana (Schott) Christenh. | China, Xianhu, XHLZ001 | *C_AA120346.1 |
| Crypsinoideae | Drynaria mollis Bedd. | China, Yunnan, YYH19492 | *C_AA120348.1 |
| Crypsinoideae | Drynaria parishii (Bedd.) Bedd. | China, X.C. Zhang 7946 | MW876315 |
| Crypsinoideae | Drynaria propinqua (Wall. ex Mett.) J.Sm. ex Bedd. | China,LANKE, F14614 | *C_AA120343.1 |
| Crypsinoideae | Drynaria propinqua (Wall. ex Mett.) J.Sm. ex Bedd. | China,Tibet, YYH20310 | *C_AA120354.1 |
| Crypsinoideae | Drynaria propinqua (Wall. ex Mett.) J.Sm. ex Bedd. | China,Tibet, YYH23453 | *C_AA120357.1 |
| Crypsinoideae | Drynaria propinqua (Wall. ex Mett.) J.Sm. ex Bedd. | China,Tibet, YYH23471 | *C_AA120358.1 |
| Crypsinoideae | Drynaria quercifolia (L.) J.Sm. | China, Xianhu, F0051645 | *C_AA120340.1 |
| Crypsinoideae | Drynaria quercifolia (L.) J.Sm. | China, Xianhu, F0051725 | *C_AA120341.1 |
| Crypsinoideae | Drynaria quercifolia (L.) J.Sm. | - | MT130596 |
| Crypsinoideae | Drynaria quercifolia (L.) J.Sm. | China, Yunnan, YYH19409 | *C_AA120347.1 |
| Crypsinoideae | Drynaria rigidula (Sw.) Bedd. | China,LANKE, CXH006 | *C_AA120353.1 |
| Crypsinoideae | Drynaria rigidula (Sw.) Bedd. | China, Xianhu, F0034038 | *C_AA120338.1 |
| Crypsinoideae | Drynaria rigidula (Sw.) Bedd. | China, Hainan, YYH20591 | *C_AA120355.1 |
| Crypsinoideae | Drynaria roosii Nakaike | China, Hainan, CXH001 | *C_AA120333.1 |
| Crypsinoideae | Drynaria roosii Nakaike | China, Guangdong, CXH004 | *C_AA120335.1 |
| Crypsinoideae | Drynaria roosii Nakaike | China, Fujian, SJP00016 | *C_AA120344.1 |
| Crypsinoideae | Drynaria roosii Nakaike | China, Fujian, YYH22185 | *C_AA120350.1 |
| Crypsinoideae | Drynaria speciosa (Blume) Christenh. | Indonesia, Java, R. Wei 508 | MW876316 |
| Crypsinoideae | Drynaria speciosa (Blume) Christenh. | China, Xianhu, XHDY | *C_AA120344.1 |
| Crypsinoideae | Drynaria willdenowii (Bory) T. Moore | Madagascar, X.C. Zhang 9062 | MW876317 |
| Crypsinoideae | Selliguea albidoglauca (C. Chr.) S.G. Lu, Hovenkamp & M.G. Gilbert | China, Yunnan, X.C. Zhang 7609 | MW876360 |
| Crypsinoideae | Selliguea capitellata (Wall. ex Mett.) X.C. Zhang & L.J. He | China, Yunnan, X.C. Zhang 6341 | MW876361 |
| Crypsinoideae | Selliguea chenopus (Christ) S.G. Lu, Hovenkamp & M.G. Gilbert | China, Yunnan, X.C. Zhang 6259 | MW876366 |
| Crypsinoideae | Selliguea trilobus (Houtt.) M. G. Price | China, Yunnan, YYH20789 | *C_AA120356.1 |
| Crypsinoideae | Selliguea yakushimensis (Makino) Fraser-Jenk. | - | MN623352 |
| Crypsinoideae | Synammia feuillei (Bertero) Copel. | Chile, Lendemer 16329 | MW876374 |
| Crypsinoideae | Synammia intermedia (Colla) G. Kunkel | Chile, Juan Fernandez Isl., C. Scottsberg 432 | MW876375 |
| Grammitidoideae | Micropolypodium sikkimense (Hieron.) X.C. Zhang | China, Xizang, Z.Y. Li 2763 | MW876339 |
| Grammitidoideae | Oreogrammitis congener (Blume) Parris | Indonesia, Java, R. Wei et al., 523 | MW876347 |
| Grammitidoideae | Prosaptia khasyana (Hook.) C. Chr. & Tardieu | China, Guangxi, X.C. Zhang 6038 | MW876356 |
| Loxogrammoideae | Dictymia brownii Copel. | Australia, R. Coveny 10858 | MW876312 |
| Loxogrammoideae | Loxogramme lankokiensis (Rosenst.) C.Chr. | - | MW039342 |
| Microsoroideae | Bosmania membranacea (D.Don) Testo | China, Yunnan, R. Wei WR0368 | MW876309 |
| Microsoroideae | Goniophlebium niponicum (Mett.) Bedd. | China, SS Liu 20161013 | NC_040221 |
| Microsoroideae | Microsorum cuspidatum (D.Don) Tagawa | - | NC_056108 |
| Microsoroideae | Thylacopteris papillosa (Blume) J. Sm. | Indonesia, Java, R. Wei 345 | MW876376 |
| Platycerioideae | Hovenkampia schimperiana (Mett.) Li Bing Zhang & X.M.Zhou | Kenya, B. Liu CPG27233 | MW876325 |
| Platycerioideae | Platycerium bifurcatum (Cav.) C.Chr. | - | MN623367 |
| Platycerioideae | Pyrrosia bonii (Christ ex Giesenh.) Ching | China, SS Liu 201615 | MH352390 |
| Platycerioideae | Pyrrosia subfurfuracea (Hook.) Ching | - | NC_047436 |
| Polypodioideae | Pecluma dulcis (Poir.) F.C. Assis & Salino | Lehtonen and Cárdenas (2019) | MK705755 |
| Polypodioideae | Pleopeltis macrocarpa (Bory ex Willd.) Kaulf. | Kenya, H. Shang SG1191 | MW876352 |
| Polypodioideae | Pleurosoriopsis makinoi (Maxim. ex Makino) Fomin | China, Sichuan, X.C. Zhang 9478 | MW876354 |
| Polypodioideae | Polypodium sibiricum Sipliv. | China, Heilongjiang, X.C. Zhang 7312 | MW876355 |
| Serpocauloideae | Serpocaulon fraxinifolium (Jacq.) A.R. Sm. | Brazil, G. Hatschbach et al. 73838 | MW876370 |

## Supplementary Table S2． Basic features of *Drynaria* plastomes.

| Species | Size/bp | | | | GC content/% | | | |
| --- | --- | --- | --- | --- | --- | --- | --- | --- |
|  | Genome | LSC | SSC | IR | Genome | LSC | SSC | IR |
| *D. coronans* | 161196 | 80816 | 21406 | 29487 | 41.30 | 39.50 | 36.80 | 45.50 |
| *D.speciosa* | 151473 | 80661 | 21568 | 24622 | 40.80 | 39.50 | 36.80 | 44.60 |
| *D. heraclea* | 152772 | 80542 | 21636 | 25297 | 41.00 | 39.60 | 36.80 | 44.90 |
| *D. meyeniana* | 157008 | 80677 | 27101 | 24615 | 41.20 | 39.50 | 40.20 | 44.60 |
| *D. propinqua* | 152514 | 80967 | 21543 | 25002 | 40.80 | 39.50 | 37.00 | 44.60 |
| *D. parishii* | 163438 | 80953 | 21553 | 30466 | 41.50 | 39.50 | 37.00 | 45.70 |
| *D. roosii* | 154010 | 85741 | 21429 | 23420 | 40.90 | 39.80 | 36.60 | 45.10 |
| *D. baronii* | 151626 | 83206 | 21624 | 23398 | 40.80 | 39.40 | 37.00 | 45.00 |
| *D. delavayi* | 151698 | 80921 | 21614 | 24581 | 40.80 | 39.60 | 37.00 | 44.40 |
| *D. mollis* | 151617 | 80832 | 21623 | 24581 | 40.80 | 39.60 | 37.00 | 44.40 |
| *D. bonii* | 151582 | 80760 | 21634 | 24594 | 40.60 | 39.30 | 36.60 | 44.50 |
| *D. quercifolia* | 151578 | 80752 | 21648 | 24589 | 40.60 | 39.30 | 36.60 | 44.40 |
| *D. descensa* | 151543 | 80723 | 21642 | 24589 | 40.60 | 39.30 | 36.70 | 44.50 |
| *D. rigidula* | 153698 | 80731 | 23751 | 24608 | 40.80 | 39.50 | 37.80 | 44.50 |
| *D. willdenowii* | 153821 | 82063 | 21598 | 25080 | 41.00 | 39.70 | 36.80 | 45.00 |

## Supplementary Table S3． Basic features of *Drynaria* plastomes.

| Gene function | Gene type | Name of gene |
| --- | --- | --- |
| Photosynthesis | Subunits of photosystem I | *psa*B,*psa*A,*psa*I,*psa*J,*psa*C |
|  | Subunits of photosystem II | *psb*K,*psb*I,*psb*M,*psb*Z,*psb*C,*psb*D,*psb*J,*psb*L,*psb*F,*psb*E,*psb*B,*psb*T,*psb*N,*psb*H,*psb*A(2) |
|  | Subunits of NADH dehydrogenase | *ndh*B*(3)*,*ndh*J,*ndh*K,*ndh*C,*ndh*F,*ndh*D,*ndh*E,*ndh*G,*ndh*I,*ndh*A,*ndh*H |
|  | Subunits of cytochrome b/f complex | *pet*N,*pet*A,*pet*L,*pet*G,*pet*B,*pet*D |
|  | Subunits of ATP synthase | *atp*A,*atp*F,*atp*H,*atp*I,*atp*E,*atp*B |
|  | Large subunit of rubisco | *rbc*L |
|  | Subunits photochlorophyllide reductase | *chl*B,*chl*N,*chl*L |
| Self-replication | Proteins of large ribosomal subunit | *rpl*33,*rpl*20,*rpl*36,*rpl*14,*rpl*16,*rpl*22,*rp*l2,*rpl*23,*rpl*21,*rpl*32 |
|  | Proteins of small ribosomal subunit | *rps*16,*rps*2,*rps*14,*rps*4,*rps*18,*rps*12(3),*rps*11,*rps*8,*rps*3,*rps*19,*rps*7(2),*rps*15 |
|  | Subunits of RNA polymerase | *rpo*C2,*rpo*C1,*rpo*B,*rpo*A |
|  | Ribosomal RNAs | *rrn*5(2),*rrn*4.5(2),*rrn*23(2),*rrn*16(2) |
|  | Transfer RNAs | *trn*Q*-*UUG,*trn*S-GCU,*trn*G*-*UCC,*trn*R-UCU,*trn*D-GUC,*trn*Y-GUA,*trn*E-UUC,*trn*C-GCA,*trn*G-GCC,*trn*S-UGA,*trn*T-GGU,*trn*fM-CAU,*trn*S-GGA,*trn*L-UAA,*trn*F-GAA,*trn*V-UAC,*trn*M-CAU,*trn*R-UCG,*trn*W-CCA,*trn*P-UGG,*trn*I-CAU,*trn*T-UGU(2),*trn*R-ACG(2),*trn*A-UGC(2),*trn*I-GAU(2),*trn*H-GUG(2),*trn*N-GUU(2),*trn*P-GGG,*trn*L-UAG |
| Other genes | Maturase | *mat*K |
|  | Protease | *clp*P |
|  | Envelope membrane protein | *cem*A |
|  | Acetyl-CoA carboxylase | *acc*D |
|  | c-type cytochrome synthesis gene | *ccs*A |
|  | Translation initiation factor | *inf*A |
| Nnknown function | Open reading frame | *ycf*1,*ycf*2,*ycf*3,*ycf*4 |
